# Cardiorespiratory Anomalies in Mice Lacking CB_1_ Cannabinoid Receptors

**DOI:** 10.1371/journal.pone.0100536

**Published:** 2014-06-20

**Authors:** Alessandro Silvani, Chiara Berteotti, Stefano Bastianini, Gary Cohen, Viviana Lo Martire, Roberta Mazza, Uberto Pagotto, Carmelo Quarta, Giovanna Zoccoli

**Affiliations:** 1 PRISM Lab, Department of Biomedical and Neuromotor Sciences, Alma Mater Studiorum – University of Bologna, Bologna, Italy; 2 Department of Women & Child Health, Karolinska Institutet, Stockholm, Sweden; 3 Endocrinology Unit and Center for Applied Biomedical Research, Department of Medical and Surgical Sciences, S. Orsola University Hospital, Alma Mater Studiorum – University of Bologna, Bologna, Italy; INSERM, France

## Abstract

Cannabinoid type 1 (CB_1_) receptors are expressed in the nervous and cardiovascular systems. In mice, CB_1_ receptor deficiency protects from metabolic consequences of a high-fat diet (HFD), increases sympathetic activity to brown fat, and entails sleep anomalies. We investigated whether sleep-wake and diet-dependent cardiorespiratory control is altered in mice lacking CB_1_ receptors. CB_1_ receptor knock-out (KO) and intact wild-type (WT) mice were fed standard diet or a HFD for 3 months, and implanted with a telemetric arterial pressure transducer and electrodes for sleep scoring. Sleep state was assessed together with arterial pressure and heart rate (home cage), or breathing (whole-body plethysmograph). Increases in arterial pressure and heart rate on passing from the light (rest) to the dark (activity) period in the KO were significantly enhanced compared with the WT. These increases were unaffected by cardiac (β_1_) or vascular (α_1_) adrenergic blockade. The breathing rhythm of the KO during sleep was also more irregular than that of the WT. A HFD increased heart rate, impaired cardiac vagal modulation, and blunted the central autonomic cardiac control during sleep. A HFD also decreased cardiac baroreflex sensitivity in the KO but not in the WT. In conclusion, we performed the first systematic study of cardiovascular function in CB_1_ receptor deficient mice during spontaneous wake-sleep behavior, and demonstrated that CB_1_ receptor KO alters cardiorespiratory control particularly in the presence of a HFD. The CB_1_ receptor signaling may thus play a role in physiological cardiorespiratory regulation and protect from some adverse cardiovascular consequences of a HFD.

## Introduction

Endogenous cannabinoids, such as arachidonoylethanolamide (anandamide) and 2-arachidonoylglycerol (2-AG), play contrasting roles in cardiovascular regulation [1]. For example, endocannabinoids decrease cardiac contractility and lower arterial pressure in a model of genetic hypertension [2], and contribute to vasodilation during hemorrhagic shock [3]. These effects are mediated by cannabinoid type 1 (CB_1_) receptors, which are expressed in the heart and blood vessels, sympathetic ganglia, and throughout the brain [4], [5].

The endocannabinoid system in the brain [6] and non-neural peripheral tissues is modulated by diet [7]. We have shown that lack of CB_1_ receptors protects from adverse metabolic consequences of a high-fat diet (HFD), e.g. visceral obesity and high plasma glucose, cholesterol, and triglycerides [5]. These protective effects are associated with increased sympathetic activation of brown adipose tissue, which burns fat to produce heat [5]. More recently, we have found that CB_1_ receptor blockade during fasting causes hypophagia by increasing sympathetic activity to the gastrointestinal system and, as a result, afferent activation of the nucleus of the solitary tract in the medulla [8]. However, it remains unclear whether deficiency of CB_1_ receptor signaling increases sympathetic activation of cardiovascular effectors as well as brown fat and the gastrointestinal system. If so, absence of CB_1_ receptors may offer a measure of metabolic “protection” from a HFD, while at the same time paradoxically worsening the cardiovascular derangements provoked by HFD and obesity [9], [10]. Furthermore, cardiovascular control under conditions of defective CB_1_ receptor signaling may be compromised by sleep-related behavioral and respiratory anomalies. For instance, absence of CB_1_ receptors is associated with multiple sleep-related anomalies and greater arousal during the active period of the day [11], and systemic cannabinoid administration seems to stabilize breathing rhythm during sleep [12].

In this study, we tested the hypothesis that absence of CB_1_ receptor signaling disturbs wake-sleep behavior- and diet-dependent cardiovascular and respiratory control. We present the first detailed, simultaneous evaluation of the cardiovascular, respiratory and wake-sleep phenotype of freely-behaving CB_1_ receptor knock-out mice (KO) [13]. The KO and wild-type (WT) control mice were fed either a standard diet (SD) or HFD for 4 months.

## Methods

The study was carried out in accordance with the recommendations in the Guide for the Care and Use of Laboratory Animals of the National Institutes of Health. The protocol was approved by the Committees on the Ethics of Animal Experiments of the University of Bologna and of the Italian Ministry of Education, University, and Research (Permit Number: 8137). All surgery was performed under isoflurane anesthesia (1.8–2.4% in O_2_) with intra-operative analgesia (Carprofen 0.1 mg s.c., Pfizer Italy, Latina), and all efforts were made to minimize discomfort and distress.

### Mice

Experiments were performed on male WT mice and male KO mice with a congenital deficiency of CB_1_ receptors (i.e. homozygosis for the Cnr1tm1.1Ltz allele of the Cnr1 gene) [13]. All mice were congenic (back-crossed 7 generations to C57Bl/6N), maintained at the laboratory animal facilities of the University of Bologna, Italy, and genotyped by polymerase-chain reaction as previously described [5], [13].

### Diets

The breeders and weaned pups were fed a mouse SD (12.3 KJ/g: 11% fat, 19% proteins, 70% carbohydrates; laboratory of Dr. Piccioni, Gessate, Milano, Italy) [5], [11]. At age 8 weeks, mice for study were randomly placed on a HFD (18.9 KJ/g: 40% fat, 15% proteins, 45% carbohydrates; laboratory of Dr. Piccioni) [5], [11] or maintained on a SD for >15 weeks until the termination of the experiment, yielding 4 experimental groups: WT-SD (n = 9), KO-SD (n = 10), WT-HFD (n = 10), KO-HFD (n = 9). Data describing sleep alterations associated with CB_1_ receptor deficiency have previously been published for these animals [11]. In the present study, an additional group of KO-HFD mice (n = 6) was included and tested pharmacologically during the light and the dark periods to assess cardiovascular activation during the light-dark transition (LDT) under conditions of sympathetic receptor blockade.

### Surgery

Mice were instrumented with electrodes for electroencephalography (EEG) and neck muscle electromyography (EMG) recordings. A calibrated telemetric arterial pressure transducer (TA11PAC10, Data Science International, Tilburg, The Netherlands) was implanted subcutaneously, and the catheter tip advanced via the femoral artery until it lay in the abdominal aorta below the renal arteries. Surgical procedures followed a published protocol [14]. Mouse age at surgery averaged 20.1±0.3 weeks (mean ± SEM) and did not differ significantly between groups. Mouse weight at surgery was significantly lower for the KO (SD, 25.6±0.8 g; HFD, 27.2±0.9 g) versus the WT (SD, 29.0±1.5 g; HFD, 30.8±0.9 g). A detailed analysis of body weight of these mice throughout the dietary treatment has been recently published [11].

### Experimental protocol

After surgery, mice were housed individually and allowed 12–15 days to recover. Simultaneous sleep and breathing recordings were then made inside a whole-body plethysmograph for 8 hours, starting at the onset of the light period (i.e, Zeitgeber Time 0, ZT0). After further 2–5 days recovery, mice underwent undisturbed 48 hour baseline recordings of sleep and arterial pressure in their cages. Mice then underwent a sequence of tests to evaluate sleep control (6 hours of sleep deprivation, 18 hours of sleep recovery, 6 hours of cage switch), which have been described in detail in a previous publication [11]. After a further 24 hours recovery, mice received intra-peritoneal (ip) bolus injections of prazosin (P7791, Sigma Aldrich, St. Louis, MO, USA), which blocks the α_1_ adrenergic receptors that mediate sympathetic vasoconstriction, and metoprolol (M5391, Sigma Aldrich), which blocks the β_1_ adrenergic receptors that mediate sympathetic increases in heart rate and contractility. Drugs were administered in random order on 2 successive days during the light period (ZT6-ZT12) whilst sleep state and arterial pressure were continuously recorded. On the second day, mice also received a control ip injection of saline at ZT0-ZT6 (i.e., 6 hours before the drug was injected). The volume of all injections was 10 µL/g. Prazosin (dose: 1 µg/g) was dissolved in 5% dextrose-5% glycerol in saline [15]. Metoprolol (dose: 4 µg/g) was dissolved in saline [15]. All recordings were performed under a 12∶12-h light-dark cycle with lights on (ZT0) at 09:00, at an ambient temperature of 25°C, with free access to water and food.

A separate group of 6 KO mice fed a HFD was injected ip with saline, prazosin and metoprolol at the above doses, randomly at the end of the light period (ZT6-ZT12) as well as under dim light at the beginning of the dark period (ZT12-ZT18), whilst sleep and arterial pressure were continuously recorded. Each mouse received 2–5 injections of each drug during the light period and the same during the dark period. Injections were separated by a minimum 18 hour washout period. To facilitate injecting in the dark, this group of mice was habituated to a different light schedule (ZT0 at 03:00; continuous dim light during the dark period) from the time of surgery.

### Data acquisition

The EEG, EMG and breathing of mice unrestrained except for the electrode tether was recorded continuously inside a modified 2-chamber whole-body plethysmograph (PLY4223, Buxco, Wilmington, NC, USA). The mouse chamber (volume 0.97 L) accommodated a rotating electrical swivel (SL6C/SB, Plastics One, Roanoke, VA, USA) and probes to measure temperature and humidity (PC52-4-SX-T3 sensor, Rense Instruments, Rowley, MA, USA). The differential pressure between the two plethysmograph chambers was measured with a high-precision pressure transducer (DP103-06, Validyne Engineering, Northridge, CA, USA). The chamber was continuously purged with air at a relatively high flow rate (1.5 L/min) to prevent CO_2_ build-up. The system was calibrated dynamically with a 100 µL micro-syringe (Hamilton, Reno, NV, USA) at the termination of each recording, substituting the mouse with an object of similar volume [16], [17].

Baseline recordings and drug challenges were performed on freely-behaving mice housed individually in cages with simultaneous acquisition of the EEG, EMG, and arterial pressure, as previously described [14]. The EEG and EMG signals were transmitted via a cable connected to a rotating swivel (SL2+2C/SB, Plastics One) on a balanced suspensor arm. The arterial pressure signal was transmitted telemetrically via radio waves to a receiver located under the cage.

The signals were synchronized during analog-to-digital conversion [14] and digitized at 16-bit and 1024 Hz using acquisition hardware (National Instruments, Austin, TX, USA) and custom-written software (Labview, National Instruments). The signals other than arterial pressure were down-sampled for data storage (EEG, EMG, and plethysmograph pressure at 128 Hz; plethysmograph temperature and humidity at 4 Hz).

### Data analysis

Data analysis was performed with MatLab (Mathworks, Natick, MA, USA). Scoring of wakefulness, non-rapid-eye-movement sleep (NREMS), and rapid-eye-movement sleep (REMS) was performed visually by scrolling through raw EEG and EMG records at 4-s resolution, as previously described [14].

Beat-to-beat mean arterial pressure (MAP), systolic arterial pressure (SAP), heart rate (HR) and heart period (HP) were computed from the raw arterial pressure signal as previously described [14]. Differences between the average MAP and HR values in the first 6 hours of the dark period (ZT12-ZT18) and those in the last 6 hours of the light period (ZT6-ZT12) were computed to assess cardiovascular activation across the LDT.

For pharmacological experiments, mean MAP and HR during wakefulness was computed 0.75–1.25 hours post-injection, once arousal effects of the injection had subsided [15]. Cardiovascular values post-injection were compared with baseline (the 1.5 hours before injection). For technical reasons, data post-prazosin injection during the light period were not obtained for 2 WT-SD, 3 KO-SD, 1 WT-HFD, and 3 KO-HFD within the main group of mice treated pharmacologically during the light period. Data post-prazosin and saline injections were also not available for 1 KO-HFD mouse from the additional group treated across the LDT. The main problem was that 10/74 prazosin and 2/62 metoprolol injections were followed by a dramatic fall in HR below 300 bpm, possibly a reflexive vagal reaction. These extreme events occurred for mice of both genotypes and dietary treatment groups. Consequently, these animals were excluded from the analysis.

Baseline cardiac baroreflex sensitivity (BRS) was computed from SAP and HP using the sequence technique. The amplitude of cardiac vagal modulation was estimated with the index pNN8, which measures the percentage of HP values that differ from the immediately succeeding HP by >8 ms. The contributions of the baroreflex and central autonomic commands to cardiac control were assessed by computing HP vs. SAP cross-correlation functions (CCF), and by coherent averaging of spontaneous SAP surges from artefact-free wake-sleep episodes of >60 s, following published protocols [18].

Technically satisfactory plethysmograph recordings were obtained and analyzed for 7 WT-SD, 5 WT-HFD, 4 KO-SD, and 8 KO-HFD mice. Breathing analysis was performed on stable wake-sleep episodes lasting ≥12 s (i.e., at least 3 consecutive 4-s epochs). Individual breaths were identified automatically from the upward (+) plethysmograph pressure deflection peak. Errors in breath detection as well as pressure artefacts (e.g., due to movements) were manually excluded from the analyses. Stable, artefact-free periods of breathing comprised 74±2% and 59±4% of the NREMS and REMS recordings, respectively, but only 7±2% of the time awake. A detailed analysis of breathing was therefore confined to periods of sleep.

Instantaneous total breath duration (i.e. the interval between successive breaths, T_TOT_), tidal volume (V_T_) and minute volume (V_E_ = V_T_/T_TOT_) were calculated, and volumes were expressed per gram body weight [16], [17], [19]. For each mouse in each sleep state, augmented breaths (sighs) were defined as V_T_>3 times average V_T_, and apneas were defined as T_TOT_>3 times average T_TOT_.

The short-term and long-term variability of T_TOT_ was estimated from Poincaré plots of the T_TOT_ of consecutive breaths [20]. The standard deviations of T_TOT_ around a new set of axes oriented with (SD_1_) or orthogonal to (SD_2_) the line of identity of the Poincaré plots were calculated using published formulas to estimate the short-term and long-term variability of T_TOT_, respectively [21].

### Statistics

Statistical tests were performed with SPSS (SPSS, Chicago, IL, USA) and significance at *P*<0.05. Data are reported as means ± SEM and were analysed by ANOVA (GLM procedure with Huynh-Feldt correction when appropriate) to test for main effects of genotype (2 levels: KO and WT) and diet (2 levels: SD and HFD) and interaction effects. In case of significance of diet x genotype interaction effects, differences between groups of mice were tested by t-tests with four planned comparisons (KO-SD vs. WT-SD, WT-HFD vs. WT-SD, KO-HFD vs. KO-SD, and KO-HFD vs. WT-HFD).

## Results

### Daily rhythms of arterial pressure and heart rate

**Figure 1:**
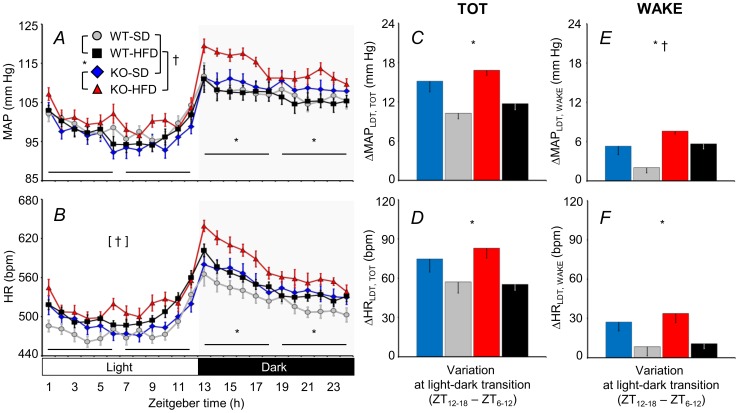
Daily profiles of mean arterial pressure and heart rate. Panels A and B show the daily profiles of mean arterial pressure (MAP) and heart rate (HR). Zeitgeber time (ZT) is time from lights on. The other panels show differences (Δ) in MAP and HR across the light-dark transition (LDT), i.e., between the first six hours of the dark period (ZT12 - ZT18) and the last 6 hours of the light period (ZT6 - ZT12). Panels C and D show data computed regardless of the wake-sleep state (TOT) or exclusively during epochs of wakefulness (WAKE). Data are means ± SEM for cannabinoid type 1 receptor knock-out mice (KO) and wild-type (WT) mice fed a standard diet (SD) or a high-fat diet (HFD), with n = 9–10 per group. The statistical analysis in panels A and B was performed on the average values over 6-hour periods (horizontal lines). * and †, *P*<0.05, main effects of genotype (KO vs. WT) and diet (HFD vs. SD), respectively. Symbols within brackets indicate significant main effects (ANOVA). Detailed ANOVA results are reported in Table S1.

The MAP and HR were both significantly higher for KO versus WT, but only during the dark (active) period (Figure 1A,B; cf. Table S1 for statistical detail). Thus, increases in MAP and HR across the LDT were significantly greater for the KO compared with the WT (Figure 1C–D). This effect of genotype was weaker but remained statistically significant when the increases in MAP and HR across the LDT were only computed for epochs of wakefulness (Figure 1E–F).

The 24-h average HR was higher for mice fed a HFD compared with those fed a SD (Figure 1B). The increase in MAP computed for epochs of wakefulness across the LDT was also enhanced by a HFD (Figure 1E).

### Effects of adrenergic receptor blockade

**Figure 2:**
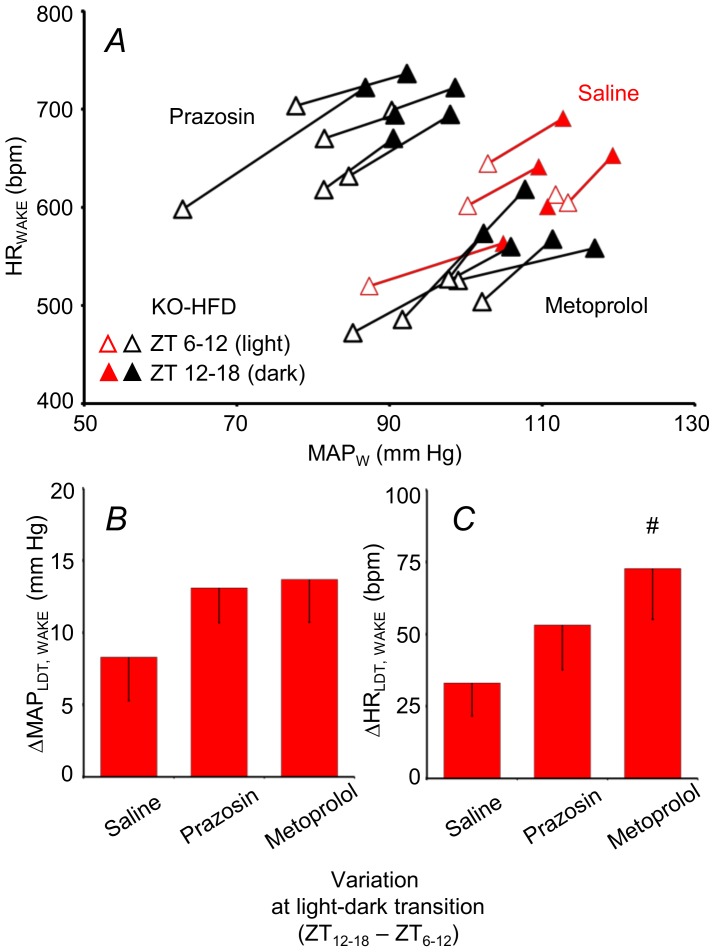
Cardiovascular effects of adrenergic receptor blockade. Panel A shows mean values of MAP and HR during WAKE in a group of KO mice fed a HFD and subjected to pharmacological treatment with saline (vehicle, n = 5), prazosin (a α_1_ adrenergic receptor blocker, n = 6) and metoprolol (a β_1_ adrenergic receptor blocker, n = 5) during both the light and dark periods. Segments connect the values measured in the same mouse after drug or vehicle injection during the light (open triangles) and dark (filled triangles) periods. Values of MAP and HR after prazosin and metoprolol formed two separate clusters and are shown in black and white. Values of MAP and HR after saline are shown in red and white. Panels B and C show means and SEM of ΔMAP and ΔHR during WAKE across the light-dark transition (LDT) for the same mice in A. #, *P*<0.05, vs. saline. For other abbreviations, see Figure 1.

The values of MAP and HR between 0.75 and 1.25 hours after saline (vehicle) injection did not differ significantly from those in the 1.5 hours before injection. In the same time window, blockade of α_1_ vascular adrenergic receptors with prazosin and blockade of β_1_ cardiac adrenergic receptors with metoprolol significantly reduced MAP and HR during wakefulness in the light period, and the extent of these reductions did not differ significantly between groups (Figure S1). To investigate the effects of adrenergic receptor blockade on the cardiovascular activation across the LDT, we subjected a separate group of KO mice fed a HFD to prazosin and metoprolol injections at the end of the light period and at the beginning of the dark period. As expected, prazosin decreased MAP and reflexively increased HR of these mice, whereas metoprolol reduced both MAP and HR (Figure 2A). However, the increase in MAP across the LDT was not blunted either by prazosin or metoprolol, whereas the increase in HR across the LDT was actually enhanced by metoprolol compared with saline (Figure 2B,C).

### Cardiovascular changes as a function of the wake-sleep state

**Figure 3:**
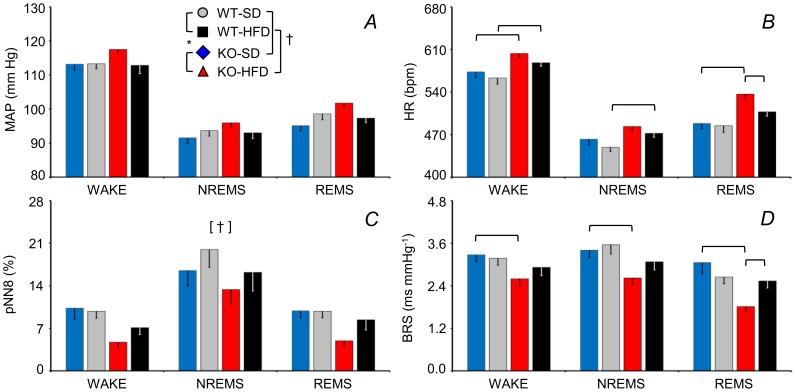
Sleep-related changes in arterial pressure, heart rate, cardiovagal modulation, and cardiac baroreflex sensitivity. Panels A-D show the mean values of MAP and HR, pNN8 (an index of cardiac vagal modulation), and spontaneous cardiac baroreflex sensitivity (BRS) during epochs of WAKE, non-rapid-eye-movement sleep (NREMS) and rapid-eye-movement sleep (REMS) averaged over 48 hours including light and dark periods. Data are means ± SEM for KO and WT mice fed a SD or a HFD, with n = 9–10 per group. The [†] symbol indicates a significant main effect of diet (*P*<0.05, ANOVA). Horizontal brackets indicate significant pairwise comparisons (*P*<0.05, *t*-test). For other abbreviations and symbols see Figure 1. Detailed ANOVA results are reported in Table S2.

The analysis of MAP, HR, the cardiovagal index pNN8, and BRS as a function of the wake-sleep state over 48 hours (light and dark periods included) are shown in Figure 3 (cf. Table S2 for statistical detail). The pNN8 index was lower in mice fed a HFD than in those fed a SD (Figure 3C). Analysis of HR and BRS revealed a significant 3-way interaction effect of genotype, diet, and wake-sleep state. In particular, KO-HFD had higher HR during wakefulness and REMS and a lower BRS during each wake-sleep state than did the KO-SD. The WT-HFD had a higher HR during wakefulness and NREMS than did the WT-SD. Lastly, the KO-HFD had a higher HR and lower BRS values than did the WT-HFD. Overall, HR was significantly increased by a HFD both in the KO and in the WT, whereas BRS was significantly decreased by a HFD in the KO only.

**Figure 4:**
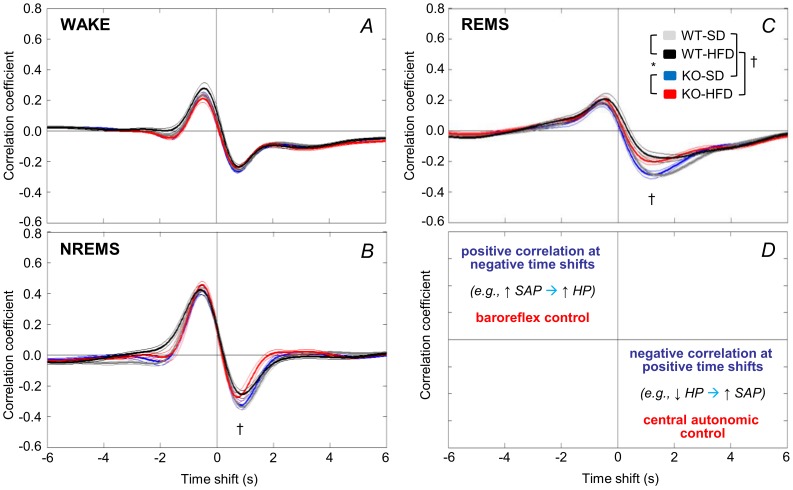
Cross-correlation analysis of cardiovascular coupling. Panels A–C show cross-correlation functions (CCF) between low-frequency (<0.8 Hz) spontaneous fluctuations of heart period (HP) and systolic arterial pressure (SAP) computed during WAKE, NREMS and REMS during 48 hours including light and dark periods. Data are means ± SEM for KO and WT mice fed a SD or a HFD, with n = 9–10 per group. Panel D summarizes the interpretation of CCF results [21]. Negative time shifts indicate that changes in HP follow SBP. A positive CCF peak at negative time shifts (e.g. cardiac slowing after SAP rises), is consistent with baroreflex buffering of SAP changes caused by fluctuations of vascular resistance. A negative CCF trough at positive time shifts (e.g. cardiac acceleration before SAP rises) is consistent with central autonomic commands acting on the heart. Statistical analysis (panels A–C) was performed on the correlation coefficients at the CCF peak (maximum positive) and trough (minimum negative) values. Detailed ANOVA results are reported in Table S3. For abbreviations and symbols see Figures 1 and 3.

The CCF between HP and SAP (Figure 4) reveals how much HP variability can be attributed to arterial baroreflex and central autonomic commands. A positive CCF peak at negative time shifts is consistent with the cardiac baroreflex response to changes in SAP that are elicited by fluctuations of vascular resistance [22]. A negative CCF trough at positive time shifts is consistent with central autonomic commands on the heart [22]. This analysis revealed a significant interaction effect between diet and wake-sleep state on the CCF trough (cf. Table S3 for statistical detail). In particular, the CCF trough during NREMS and REMS was significantly less pronounced for mice fed a HFD versus a SD.

**Figure 5:**
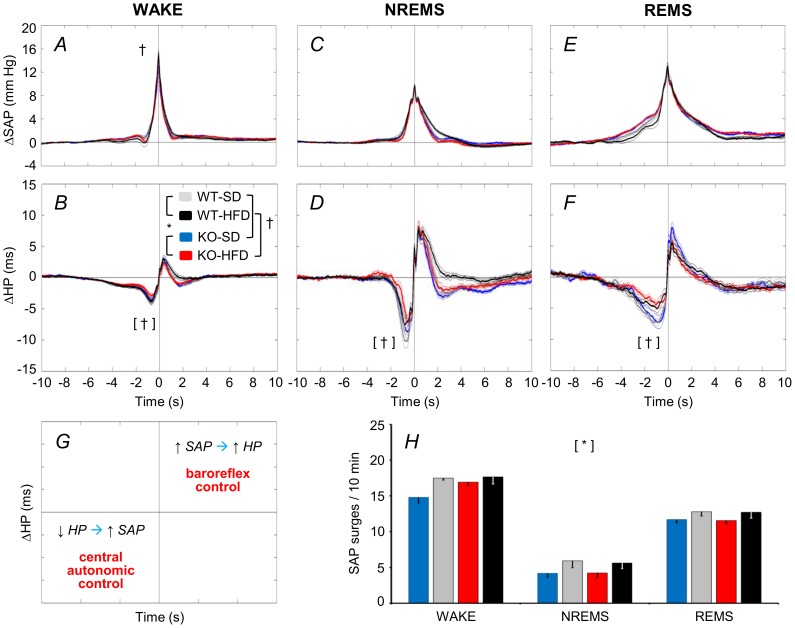
Coherent averaging of spontaneous surges of arterial pressure. The panels show time series of ΔSAP (A, C, E) and ΔHP (B, D, F) during spontaneous SAP surges (detection threshold = 5 mm Hg) during WAKE, NREMS, and REMS over 48 hours including light and dark periods. The time series were normalized by subtracting the respective baseline values and synchronized at the SAP peak. Panel G summarizes the interpretation [22]. A ΔHP trough (i.e., cardiac acceleration) preceding a SAP surge is consistent with central autonomic control of the heart. A positive ΔHP peak (i.e., cardiac slowing) following a SAP surge is consistent with baroreflex cardiac control. Statistical analysis in panels A–F was performed on ΔSAP and ΔHP peaks and the ΔHP nadir. Panel H shows the frequency of spontaneous SAP surges per 10 min of each wake-sleep state. Data are means ± SEM for KO and WT mice fed a SD or a HFD, with n = 9–10 per group. Detailed ANOVA results are reported in Table S4. For abbreviations and symbols see Figures 1 and 3.

Further insight into sleep-related changes in cardiovascular coupling is provided by coherent averaging of short-lasting SAP increases (surges) (Figure 5). These occur spontaneously in mice during each wake-sleep state. As expected from previous work on mice [18], HP decreased below baseline before SAP peaked, and increased above baseline thereafter. These HP changes are consistent with central autonomic and baroreflex control of the heart, respectively [22] (Figure 5G). The SAP surges were less frequent in KO versus WT mice (Figure 5H, cf. Table S4 for statistical detail). The HP nadir preceding the SAP peak was less pronounced for mice fed a HFD versus those fed a SD. This analysis also revealed significant interaction effects between diet and wake-sleep state on peak SAP and on the maximum HP increase that followed the SAP peak. The SAP peaked slightly but significantly higher during wakefulness for HFD (15.2±0.4 mmHg) versus SD fed mice (14.1±0.4 mmHg). The maximum HP increase after the SAP peak tended to be lower during REMS for HFD versus SD fed mice, but the difference was not significant (*P* = 0.056).

### Breathing as a function of the wake-sleep state

**Figure 6:**
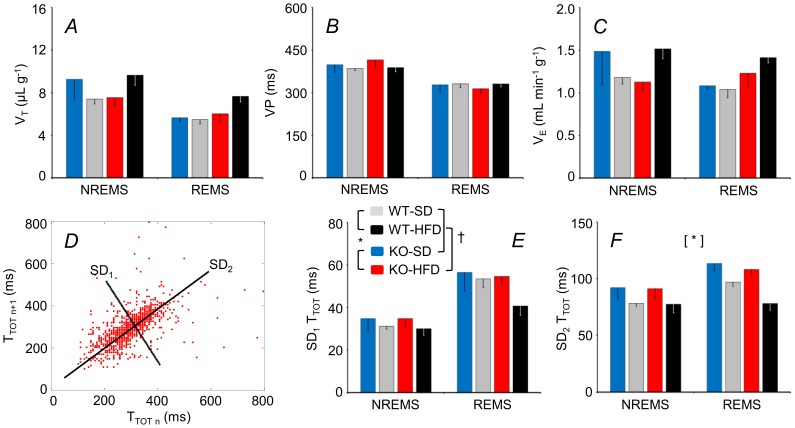
Sleep-related changes in tidal volume, respiratory period, and minute ventilation. Panels A, B, and C show tidal volume (V_T_), breath duration (T_TOT_), and minute volumes (V_E_), respectively, during light period epochs of NREMS and REMS. Panel D shows a Poincaré plot of T_TOT_ of each breath (n) versus the following breath (n+1) during REMS for a KO mouse fed a HFD. Panels E and F show the standard deviations of T_TOT_ around a new set of axes oriented with (SD_1_) or orthogonal to (SD_2_) the line of identity of the Poincaré plots, as shown in C; these reflect short-term and long-term variability of T_TOT_, respectively [20], [21]. Data are means ± SEM for KO mice fed a SD (n = 4) or a HFD (n = 8) and for WT mice fed a SD (n = 7) or a HFD (n = 5). For abbreviations and symbols see Figures 1 and 3. Detailed ANOVA results are reported in Table S5.

**Figure 7:**
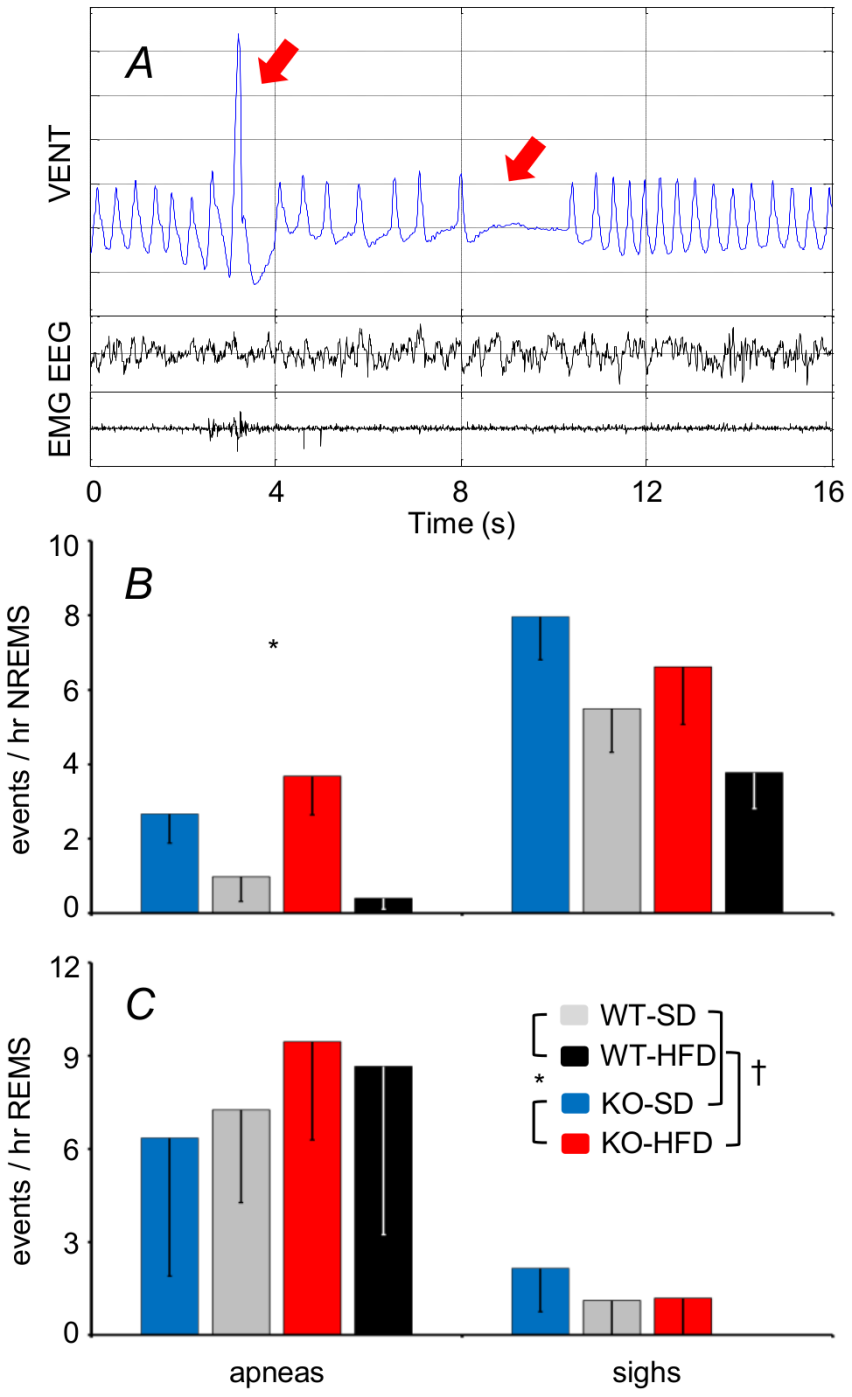
Sleep apneas and sighs. Panel A shows representative recordings of the raw plethysmograph signal (VENT), the electroencephalogram (EEG), and the neck muscle electromyogram (EMG) during NREMS for a KO mouse fed a HFD. The red arrows indicate a sigh (A; left) followed by a brief apnea (A, right). Panels B and C show the frequency of apneas (left) and sighs (right) per hour NREMS and REMS, respectively. Data are means ± SEM for KO mice fed a SD (n = 4) or a HFD (n = 8) and for WT mice fed a SD (n = 7) or a HFD (n = 5). For abbreviations and symbols see Figures 1 and 3.

Effects of genotype and diet were not significant for V_T_ or T_TOT_ (Figure 6A,B; cf. Table S5 for statistical detail) but were for V_E_ (Figure 6C). In particular, V_E_ was lower during REMS versus NREMS for the WT but not KO. Genotype significantly affected breathing variability: the KO rhythm was more irregular (SD_2_ of T_TOT_ higher) (Figure 6D–F), due to more frequent long pauses between breaths (brief apneas) during NREMS, an example of which is shown (Figure 7A,B).

## Discussion

We performed the first systematic study of cardiovascular function in CB_1_ receptor deficient KO mice during spontaneous wake-sleep behavior. Our comparison of KO and WT yielded four main novel findings. Firstly, the MAP and HR of the KO increased more on passing from the light to the dark period. Secondly, cardiac vagal modulation during different wake-sleep states was diminished by a HFD both in KO and in WT. Thirdly, a HFD attenuated BRS selectively in CB_1_ receptor KO. Finally, the KO breathing rhythm during NREMS was more irregular (long pauses between breaths were more frequent) than that of the WT.

We found that CB_1_ receptor KO significantly enhanced cardiovascular activation across the LDT (Figure 1). Regardless of genotype, mice predominately rest during the light cycle and are active during the dark [11]. The LDT for a mouse is thus equivalent to the morning rest-activity transition of a human. Humans typically exhibit a morning surge in arterial blood pressure [23]. An abnormally high early-morning blood pressure surge is associated with an increased incidence of adverse cardiovascular events such as stroke [23]. Heightened cardiovascular activity may partly explain why the peak incidence of myocardial infarction, sudden death, and stroke occurs early in the morning [24]. If, as our data suggest, CB_1_ receptors limit the morning surge in arterial pressure, they could conceivably exert a modulating influence that reduces cardiovascular risk.

We have recently provided evidence that CB_1_ receptors reduce arousal during the active period of the day [11]. The arterial pressure of a mouse is higher during the dark than during the light period for two reasons. Firstly, mice spend more time awake and less time asleep in the dark, and wakefulness increases arterial pressure. Secondly, arterial pressure within each wake-sleep state is subject to a circadian rhythm entrained to the light-dark cycle, with pressure higher during the dark period [25]. Thus, one possible explanation for the enhanced cardiovascular activity across the LDT was that the KO spent more time awake during the dark than the WT did [11]. In this respect, it is worth remarking that the effects of sleep and wakefulness on MAP were similar for KO and WT (Figure 3A), which indicates CB_1_ receptors are not essential for the central neural circuits that drive sleep-dependent changes in MAP [26]. Restricting the analysis to epochs of wakefulness revealed, however, a persistent enhancement of cardiovascular activity across the LDT of the KO (Figure 1E,F). This finding indicates that mechanisms other than altered wake-sleep behavior *per se* must enhance wakefulness cardiovascular activity during the dark in the KO.

In light of previous evidence [5], [8], we investigated whether sympathetic hyperactivity could explain the enhanced cardiovascular activity during wakefulness observed for the KO across the LDT. We focused on the α_1_ and β_1_ adrenergic receptors, which are crucial for sympathetic control of arterioles and the heart, respectively. Somewhat unexpectedly, blockade of these adrenergic receptors either did not affect or actually enhanced cardiovascular activity across the LDT of the KO-HFD (Figure 2). These data may indicate that KO cardiovascular activity is enhanced across the LDT by an actively regulated process akin to an upward shift of the MAP set-point. Blockade of either vascular or cardiac sympathetic control may then be compensated by over-activation of sympathetic effectors which remain viable, as well as by further vagal withdrawal. Alternatively, sympathetic control may not be critical for cardiovascular activation across the LDT. Immunization of humans against angiotensin II decreases early-morning arterial pressure [27], which suggests the renin-angiotensin system plays an important role in the morning BP surge [23]. Interestingly, angiotensin II induces vascular endocannabinoid release, which acts via CB_1_ receptors to attenuate the vasoconstrictor effect of angiotensin II [28]. Further experiments are needed to establish whether angiotensin II enhances LDT cardiovascular activation of the KO via this mechanism. Nonetheless, our data do not support the original hypothesis that lack of CB_1_ receptor signaling increases sympathetic activity to cardiovascular effectors, as it does to brown fat [5] and the gastrointestinal tract [8]. The activity of sympathetic efferent fibers to brown adipose tissue and to the cardiovascular system is controlled by distinct central neural pathways [29], which may be differentially modulated by the CB_1_ receptor.

Mechanisms modulating cardiac vagal activity are of great clinical interest since they appear to protect against life-threatening cardiac arrhythmias [30]. We found that HFD exerted a robust effect on pNN8 (an index of cardiac vagal modulation), which was reduced for KO and WT mice (Figure 3C). During sleep, this impairment in cardiac vagal modulation in HFD-fed mice manifested as a blunted central autonomic control of HR (Figure 4), which was associated with a blunting of the short-lasting cardiac accelerations that precede spontaneous arterial pressure surges (Figure 5B,D,F). Our finding that a HFD impaired cardiac vagal modulation contrasts with previous observations of mice with diet-induced obesity [31], but fits well with data from obese humans [9], [10] and our own data from leptin-deficient obese mice [18]. Moreover, our findings that a HFD increased the HR of both the KO and the WT mice (Figure 3B), while effects of cardiac adrenergic β_1_ receptor blockade on HR did not differ with diet (Figure S1B) suggest that a HFD also decreased cardiac vagal tone.

Obese humans exhibit reduced BRS [10]. In obese rats, reduced BRS is a result of impaired central processing of signals from the myelinated baroreflex afferents, which are tonically active at the resting (basal) arterial pressure [32]. Interestingly, we found that a HFD only reduced BRS of the KO (Figure 3D). The apparent lack of an effect of a HFD on the BRS of the WT may be due to the rather mild HFD we administered (40% calories from fat, cf. Methods). Alternatively, the KO may be more susceptible to a HFD-induced reduction in BRS because loss of CB_1_ receptor signaling decreases activity of barosensitive neurons in the nucleus of the tractus solitarius, the first central relay of the baroreflex pathway [33]. The decrease in BRS of the KO-HFD was not severe enough to decrease the baroreflex contribution to cardiac control, as shown by the CCF peak (Figure 4A–C) and the peak HP increase during BP surges (Figure 5B,D,F). A low spontaneous BRS may nevertheless be of interest since it is associated with a poor prognosis of hypertensive patients [34].

Obesity in humans tends to decrease the depth and rate of breathing (lower V_T_, T_TOT_), favoring a rapid, shallow breathing pattern that increases V_E_ [35]. Our analysis of breathing during sleep (Figure 6) yielded V_T_, T_TOT_, and V_E_ values consistent with previous work from mice [16], [36], but which did not differ as a function of diet. This may be a consequence of our relatively mild dietary stimulus, because V_E_ is reportedly increased if mice are fed a more extreme HFD [36]. The influence of genotype on V_E_ (lower in NREMS versus REMS for WT but not KO) was minor (Figure 6C). The KO breathing rhythm was, however, more irregular during NREMS (based on SD_2_ of T_TOT_, an index of *long-term* variability, Figure 6D,F) due to frequent long pauses between breaths (“apneas”; Figure 7B). This rhythm anomaly was, however, quite mild (<5 events per hour NREMS). It is unlikely to explain the associated cardiovascular anomalies: spontaneous surges in arterial pressure were actually *less* frequent for the KO (Figure 5H). Our findings do, however, suggest that endocannabinoid tonus acting via CB_1_ receptors helps to stabilize breathing, especially during NREMS. Our data fit well with evidence that cannabinoid administration to rats decreases apnea frequency during NREMS and REMS [12], and decreases the apnea-hypopnea index of patients with obstructive sleep apnea during NREMS [37]. Recent data suggest that endocannabinoids help to stabilize breathing rhythm during sleep by modulating the flow of afferent signals from the lung and airways at the level of the vagal nodose ganglion [38].

In conclusion, we provide evidence that the absence of CB_1_ receptor signaling is associated with subtle but significant alterations in sleep-wake cardiorespiratory control which are partly diet-dependent. The results thus suggest that CB_1_ receptor signaling plays a role in physiological cardiorespiratory control, and may protect from some adverse cardiovascular consequences of a HFD. In perspective, these data raise the hypothesis that treatments of the negative metabolic consequences of obesity based on long-term CB_1_ receptor blockade may have limited effectiveness in preventing obesity-related cardiovascular alterations, particularly if dietary fat content is not concomitantly reduced.

## Supporting Information

Figure S1 **Changes (Δ) of mean arterial pressure (MAP) or heart rate (HR) elicited by α_1_ (prazosin) or β_1_ (metoprolol) adrenergic receptor blockade, respectively, during wakefulness (WAKE) during the light period.** Data are means ± SEM for cannabinoid type 1 receptor knock-out mice (KO) and wild-type (WT) mice fed a standard diet (SD) or a high-fat diet (HFD), with n = 9–10 per group.
(TIF)

Table S1 **Daily profiles of mean arterial pressure and heart rate: ANOVA results.**
(DOC)

Table S2 **Cardiovascular changes as a function of the wake-sleep state: ANOVA results.**
(DOC)

Table S3 **Cross-correlation analysis of cardiovascular coupling: ANOVA results.**
(DOC)

Table S4 **Coherent averaging of spontaneous surges of arterial pressure: ANOVA results.**
(DOC)

Table S5 **Sleep-related changes in tidal volume, respiratory period, and minute ventilation: ANOVA results.**
(DOC)
