# Supplementary figures and images for: Cardiorespiratory Anomalies in Mice Lacking CB1 Cannabinoid Receptors
Source: PLoS One. 2014 Jun 20;9(6):e100536. doi: 10.1371/journal.pone.0100536 (PMC4065065; doi:10.1371/journal.pone.0100536)

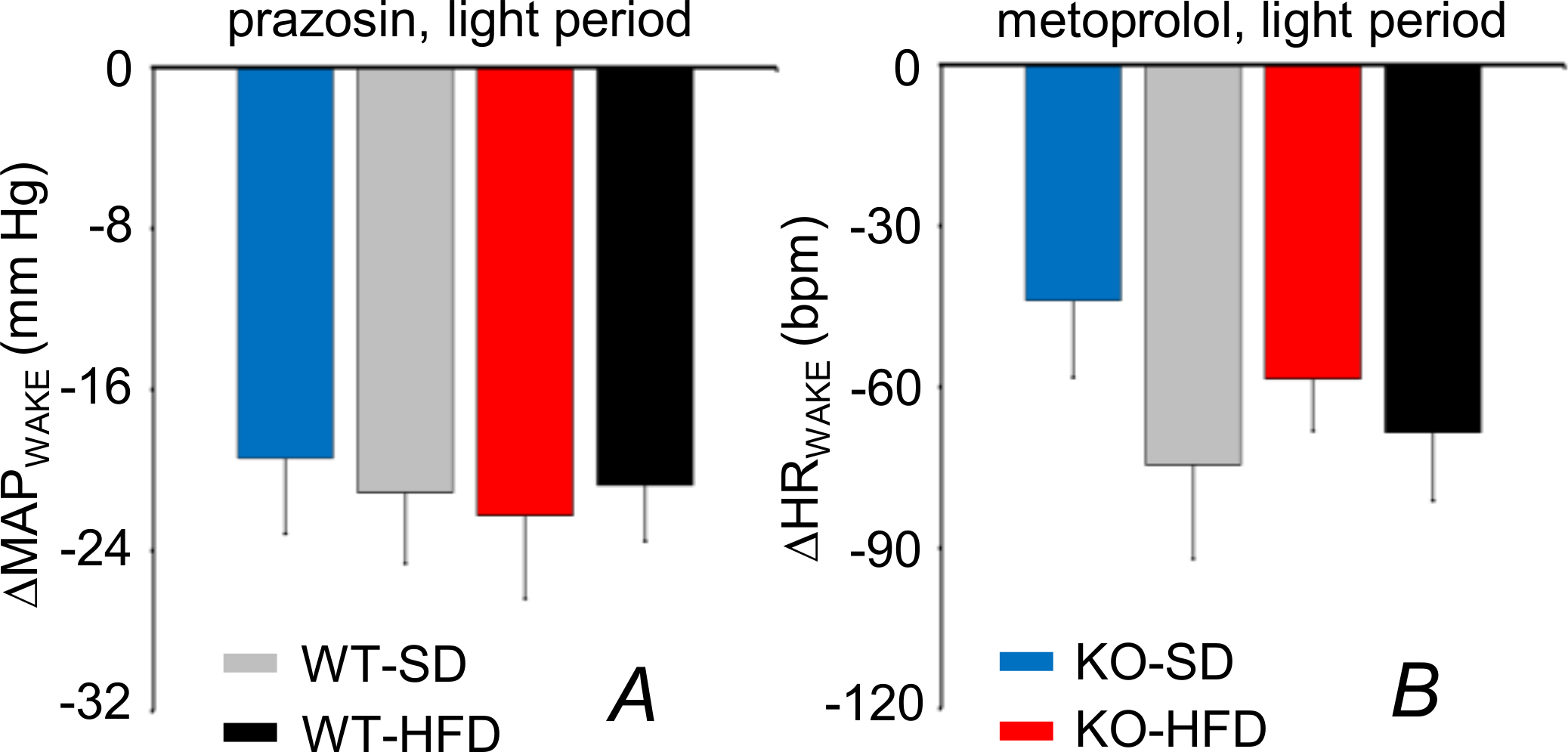

Supplement: Figure S1 — Changes (Δ) of mean arterial pressure (MAP) or heart rate (HR) elicited by α1 (prazosin) or β1 (metoprolol) adrenergic receptor blockade, respectively, during wakefulness (WAKE) during the light period. Data are means ± SEM for cannabinoid type 1 receptor knock-out mice (KO) and wild-type (WT) mice fed a standard diet (SD) or a high-fat diet (HFD), with n = 9–10 per group. (TIF) [file pone.0100536.s001.tif]
