# Supplementary material for: Cardiorespiratory Anomalies in Mice Lacking CB1 Cannabinoid Receptors
Source: PLoS One. 2014 Jun 20;9(6):e100536. doi: 10.1371/journal.pone.0100536 (PMC4065065; doi:10.1371/journal.pone.0100536)
Supplement: Table S1 — Daily profiles of mean arterial pressure and heart rate: ANOVA results. (DOC) [file pone.0100536.s002.doc]

**Table S1. Daily profiles of mean arterial pressure and heart rate: ANOVA results**

|  | **Variable** | | | |
| --- | --- | --- | --- | --- |
| **Source** | **MAP** | **HR** | **∆MAP**  **_LDT_** | **∆HR**  **_LDT_** |
| D | 0.22 | **< 0.01** | 0.10 | 0.37 |
| G | 0.12 | **0.02** | **< 0.001** | **0.04** |
| D x G | 0.08 | 0.77 | 0.78 | 0.52 |
| time | **< 0.001** | **< 0.001** |  |  |
| time x D | 0.24 | 0.13 |  |  |
| time x G | **< 0.001** | **< 0.01** |  |  |
| time x D x G | 0.56 | 0.38 |  |  |
| state |  |  | **< 0.001** | **< 0.001** |
| state x D |  |  | **0.04** | 0.92 |
| state x G |  |  | **0.04** | **< 0.01** |
| state x D x G |  |  | 0.77 | 0.67 |

Data are significance (*P*) values of the analysis of variance (ANOVA) of the hourly values of mean arterial pressure (MAP) and heart rate (HR) and of the differences (∆) in MAP and HR across the light-dark transition (LDT) for cannabinoid type 1 receptor knock-out (KO) and wild-type (WT) mice fed a standard diet (SD) or a high-fat diet (HFD), with n = 9-10 per group. The ANOVA factors were diet (D, HFD vs. SD) and genotype (G, KO vs. WT). Time (4 levels corresponding to successive 6-hour bins) was also considered as a factor for MAP and HR. State (4 levels corresponding to wakefulness, non-rapid-eye-movement sleep, rapid-eye-movement sleep, and all states merged) was also considered as a factor for ∆MAP and ∆HR. The symbol x indicates interaction effects. *P* values < 0.05 are highlighted in red for clarity. Corresponding results are reported in Figure 1.
