# Supplementary material for: Cardiorespiratory Anomalies in Mice Lacking CB1 Cannabinoid Receptors
Source: PLoS One. 2014 Jun 20;9(6):e100536. doi: 10.1371/journal.pone.0100536 (PMC4065065; doi:10.1371/journal.pone.0100536)
Supplement: Table S2 — Cardiovascular changes as a function of the wake-sleep state: ANOVA results. (DOC) [file pone.0100536.s003.doc]

**Table S2. Cardiovascular changes as a function of the wake-sleep state: ANOVA results**

|  | **Variable** | | | |
| --- | --- | --- | --- | --- |
| **Source** | **MAP** | **HR** | **pNN8** | **BRS** |
| D | 0.17 | **0.001** | **0.03** | **< 0.01** |
| G | 0.50 | 0.09 | 0.24 | 0.37 |
| D x G | 0.07 | 0.55 | 0.55 | 0.15 |
| state | **< 0.001** | **< 0.001** | **< 0.001** | **< 0.001** |
| state x D | 0.57 | 0.06 | 0.81 | 0.23 |
| state x G | 0.10 | 0.70 | 0.45 | 0.30 |
| state x D x G | 0.20 | **0.04** | 0.45 | **0.01** |

Data are significance (*P*) values of the analysis of variance (ANOVA) of mean arterial pressure (MAP), heart rate (HR), the pNN8 index of cardiovagal modulation, and the BRS index of spontaneous cardiac baroreflex sensitivity for cannabinoid type 1 receptor knock-out (KO) and wild-type (WT) mice fed a standard diet (SD) or a high-fat diet (HFD), with n = 9-10 per group. The ANOVA factors were diet (D, HFD vs. SD), genotype (G, KO vs. WT), and state (3 levels corresponding to wakefulness, non-rapid-eye-movement sleep, and rapid-eye-movement sleep). The symbol x indicates interaction effects. *P* values < 0.05 are highlighted in red for clarity. Corresponding results are reported in Figure 3.
