# Supplementary material for: Cardiorespiratory Anomalies in Mice Lacking CB1 Cannabinoid Receptors
Source: PLoS One. 2014 Jun 20;9(6):e100536. doi: 10.1371/journal.pone.0100536 (PMC4065065; doi:10.1371/journal.pone.0100536)
Supplement: Table S3 — Cross-correlation analysis of cardiovascular coupling: ANOVA results. (DOC) [file pone.0100536.s004.doc]

**Table S3. Cross-correlation analysis of cardiovascular coupling: ANOVA results**

|  | **Variable** | |
| --- | --- | --- |
| **Source** | **Peak correlation coefficient** | **Trough correlation coefficient** |
| D | 0.25 | **< 0.001** |
| G | 0.73 | 0.58 |
| D x G | 0.82 | 0.47 |
| state | **< 0.001** | **< 0.001** |
| state x D | 0.93 | **0.04** |
| state x G | 0.32 | 0.86 |
| state x D x G | 0.24 | 0.42 |

Data are significance (*P*) values of the analysis of variance (ANOVA) of the correlation coefficients at the peak and trough of cross-correlation functions between systolic arterial pressure and heart period for cannabinoid type 1 receptor knock-out (KO) and wild-type (WT) mice fed a standard diet (SD) or a high-fat diet (HFD), with n = 9-10 per group. The ANOVA factors were diet (D, HFD vs. SD), genotype (G, KO vs. WT), and state (3 levels corresponding to wakefulness, non-rapid-eye-movement sleep, and rapid-eye-movement sleep). The symbol x indicates interaction effects. *P* values < 0.05 are highlighted in red for clarity. Corresponding results are reported in Figure 4.
