# Supplementary material for: Cardiorespiratory Anomalies in Mice Lacking CB1 Cannabinoid Receptors
Source: PLoS One. 2014 Jun 20;9(6):e100536. doi: 10.1371/journal.pone.0100536 (PMC4065065; doi:10.1371/journal.pone.0100536)
Supplement: Table S4 — Coherent averaging of spontaneous surges of arterial pressure: ANOVA results. (DOC) [file pone.0100536.s005.doc]

**Table S4. Coherent averaging of spontaneous surges of arterial pressure: ANOVA results**

|  | **Variable** | | | |
| --- | --- | --- | --- | --- |
| **Source** | **∆SAP** | **∆HP peak** | **∆HP nadir** | **surges / 10 min** |
| D | 0.21 | 0.58 | **0.01** | 0.58 |
| G | 0.44 | 0.63 | 0.25 | **0.01** |
| D x G | 0.94 | 0.55 | 0.55 | 0.48 |
| state | **< 0.001** | **< 0.001** | **< 0.001** | **< 0.001** |
| state x D | **0.03** | **0.03** | 0.08 | 0.20 |
| state x G | 0.72 | 0.94 | 0.95 | 0.75 |
| state x D x G | 0.68 | 0.26 | 0.47 | 0.42 |

Data are significance (*P*) values of the analysis of variance (ANOVA) of the peak increase in systolic arterial pressure (∆SAP), the peak increase in heart period (∆HP peak), the trough decrease in heart period (∆HP nadir) during spontaneous SAP surges, and of the number of surges over 10 minutes of recordings. Data were obtained on cannabinoid type 1 receptor knock-out (KO) and wild-type (WT) mice fed a standard diet (SD) or a high-fat diet (HFD), with n = 9-10 per group. The ANOVA factors were diet (D, HFD vs. SD), genotype (G, KO vs. WT), and state (3 levels corresponding to wakefulness, non-rapid-eye-movement sleep, and rapid-eye-movement sleep). The symbol x indicates interaction effects. *P* values < 0.05 are highlighted in red for clarity. Corresponding results are reported in Figure 5.
