# Supplementary material for: Cardiorespiratory Anomalies in Mice Lacking CB1 Cannabinoid Receptors
Source: PLoS One. 2014 Jun 20;9(6):e100536. doi: 10.1371/journal.pone.0100536 (PMC4065065; doi:10.1371/journal.pone.0100536)
Supplement: Table S5 — Sleep-related changes in tidal volume, respiratory period, and minute ventilation: ANOVA results. (DOC) [file pone.0100536.s006.doc]

**Table S5. Sleep-related changes in tidal volume, respiratory period, and minute ventilation: ANOVA results**

|  | **Variable** | | | | |
| --- | --- | --- | --- | --- | --- |
| **Source** | **TV** | **T_TOT_** | **V_E_** | **SD_1_ T_TOT_** | **SD_2_ T_TOT_** |
| D | 0.11 | 0.88 | 0.09 | 0.24 | 0.24 |
| G | 0.24 | 0.61 | 0.24 | 0.08 | **< 0.01** |
| D x G | 0.20 | 0.81 | 0.29 | 0.51 | 0.63 |
| state | **< 0.001** | **< 0.001** | 0.23 | **< 0.001** | **< 0.01** |
| state x D | 0.78 | 0.68 | 0.23 | 0.27 | 0.29 |
| state x G | 0.35 | 0.08 | **0.02** | 0.52 | 0.38 |
| state x D x G | 0.79 | 0.65 | 0.51 | 0.26 | 0.41 |

Data are significance (*P*) values of the analysis of variance (ANOVA) of values of tidal volume (TV) divided by body weight, respiratory period (T_TOT_), and minute ventilation (V_E_) divided by body weight. The indexes SD_1_ and SD_2_ reflex the short-term and long-term variability of T_TOT_, respectively. Data were obtained on cannabinoid type 1 receptor knock-out (KO) mice fed a high-fat diet (HFD, n = 8) or a standard diet (SD, n = 4) and in wild-type (WT) mice fed HFD (n = 5) or SD (n = 7). The ANOVA factors were diet (D, HFD vs. SD), genotype (G, KO vs. WT), and state (non-rapid-eye-movement sleep vs. rapid-eye-movement sleep). The symbol x indicates interaction effects. *P* values < 0.05 are highlighted in red for clarity. Corresponding results are reported in Figure 6.
